# Stem Cell Conditioned Culture Media Attenuated Albumin-Induced Epithelial– Mesenchymal Transition in Renal Tubular Cells

**DOI:** 10.1159/000373984

**Published:** 2015-03-19

**Authors:** Junping Hu, Qing Zhu, Pin-Lan Li, Weili Wang, Fan Yi, Ningjun Li

**Affiliations:** a Department of Pharmacology & Toxicology, Virginia Commonwealth University School of Medicine, Richmond, VA, USA; b Department of Pharmacology, Shandong University School of Medicine, Jinan, Shandong, P.R. China

**Keywords:** E-cadherin, Fibroblast-specific protein 1, α-smooth muscle actin, NF-κB

## Abstract

**Background:**

Proteinuria-induced epithelial-mesenchymal transition (EMT) plays an important role in progressive renal tubulointerstitial fibrosis in chronic renal disease. Stem cell therapy has been used for different diseases. Stem cell conditioned culture media (SCM) exhibits similar beneficial effects as stem cell therapy. The present study tested the hypothesis that SCM inhibits albumin-induced EMT in cultured renal tubular cells.

**Methods:**

Rat renal tubular cells were treated with/without albumin (20 μmg/ml) plus SCM or control cell media (CCM). EMT markers and inflammatory factors were measured by Western blot and fluorescent images.

**Results:**

Albumin induced EMT as shown by significant decreases in levels of epithelial marker E-cadherin, increases in mesenchymal markers fibroblast-specific protein 1 and α-smooth muscle actin, and elevations in collagen I. SCM inhibited all these changes. Meanwhile, albumin induced NF-κB translocation from cytosol into nucleus and that SCM blocked the nuclear translocation of NF-κB. Albumin also increased the levels of pro-inflammatory factor monocyte chemoattractant protein-1 (MCP)-1 by nearly 30 fold compared with control. SCM almost abolished albumin-induced increase of MCP-1.

**Conclusion:**

These results suggest that SCM attenuated albumin-induced EMT in renal tubular cells via inhibiting activation of inflammatory factors, which may serve as a new therapeutic approach for chronic kidney diseases.

## Introduction

Renal tubulointerstitial fibrosis is the major underlying pathology and the common end point of progressive renal diseases and that the accumulation of myofibroblasts producing extracellular matrixes is a key element in the process of renal fibrosis [1, 2]. Emerging evidence indicates that renal tubular epithelial cells through epithelial to mesenchymal transition (EMT) is an important resource of fibrogenic myofibroblasts and plays an important role in renal tubulointerstitial fibrosis [3, 4]. During the transformation process of EMT, epithelial cells lose their specific epithelial markers and the cell-cell basement membrane contact, gain mesenchymal phenotypic changes, leading finally to spindle-shaped myofibroblasts [3, 4]. It has been reported that approximately one third of myofibroblasts are not preexisting local fibroblasts but a result of EMT [4, 5]. Therefore, EMT plays a critical role during the progression of chronic kidney damage.

Proteinuria is one of the strongest predictors for chronic kidney disease progression to end-stage renal disease [6, 7]. Evidence indicates that proteinuria may accelerate kidney disease progression to end-stage renal failure [6, 7]. Different mechanisms have been proposed for proteinuria-induced damages, including direct tubular toxicity, changes in tubular epithelial metabolism, stimulation of cytokines and chemokines and increased expression of adhesion molecules [7]. Production of proinflammatory factors by epithelial cells that are exposed to protein may be one of the major mechanisms. For example, it has been shown that protein induces the production of monocyte chemotactic protein (MCP)-1 and transforming growth factor (TGF)-β, which are the most important proinflammatory and profibrogenic factors in the progression of chronic kidney diseases [7, 8]. Proximal tubular epithelial cells that were challenged with plasma proteins secreted chemokines, such as MCP-1 and RANTES, which in return simulated the influx of mononuclear cells into the interstitium [9, 10]. Moreover, albumin, the dominant protein in proteinuria, has been shown to induce EMT, probably through stimulating the pro-inflammatory factors [11, 12]. Thus, EMT contributes to proteinuria-induced progression of chronic kidney diseases.

Stem cell therapy has been used in the treatment of different forms of diseases [13]. It has been recognized that the beneficial effects of stem cell therapy are predominantly mediated by indirect paracrine mechanisms rather than direct differentiation and substitution of damaged cells [14–16]. Conditioned media obtained from stem cell culture have been shown to improve various pathological conditions [17–19]. Administration of conditioned medium from cultured stem cells provides the same renoprotective effects as injection of stem cells [16, 20]. As the use of stem cell therapy may present some risks to the patients, stem cell conditioned media (SCM) is considered a promising alternative to stem cell therapy [21]. The present study tested the hypothesis that SCM protects against albumin-induced EMT in cultured renal tubular cells. Our results showed that SCM inhibited the albumin-induced activation of pro-inflammatory factors and blocked albumin-induced changes in EMT markers.

## Materials and Methods

### Cell culture and preparation of SCM

NRK-52E cells, a rat renal tubular cell line, were obtained from ATCC and cultured in the presence of DMEM/Ham’s F12 (DMEM/F12) medium supplemented with 10% fetal calf serum (FCS), glutamine (2 mM), penicillin (100 IU/ml), and streptomycin (100 μg/ml). Cells were cultured at 37 °C in a humidified atmosphere of 5% CO2 in air. For EMT experiments, cells were treated with 20μg/ml rat albumin (Sigma) for 48 h; for NF-κB translocation experiments, cells were treated with 20μg/ml rat albumin for 3 h.

Mesenchymal stem cells (MSCs) were generous gifts from Texas A&M Health Science Center. Passenger 7 MSCs were cultured according to the instruction in Eagle’s alpha minimum essential medium (α-MEM; Sigma), supplemented with 20% FBS (FBS, Invitrogen), 4mM L-glutamine (Invitrogen-Gibco), 100U/ml penicillin and 100ug/ml streptomycin (Invitrogen-Gibco). After 72 h, the medium was collected and used as stem cell conditioned media (SCM). The control conditioned media (CCM) were obtained from culturing rat renal medullary interstitial cells for 72 h using the same medium. Rat renal medullary interstitial cells were prepared as we described previously [22, 23]. In preliminary experiments, cells treated with CCM did not show significant difference in the EMT markers described below compared with naive cells.

### Western blot analysis

Whole cell protein and nuclear protein preparation, as well as western blotting, were performed as we described previously [24, 25]. For whole protein, the membrane was probed with primary antibodies of anti-E-cadherin (rabbit polyclonal, R&D System, 1:1000), anti-α-smooth muscle actin (SMA) (rabbit polyclonal, R&D System, 1:1000), anti-fibroblast specific protein (FSP)-1 (rabbit polyclonal, Abcam, 1:1000), anti-collagen I (rabbit polyclonal, Calbiochem, 1:1000) and anti-MCP-1 (rabbit polyclonal, Abcam, 1:500); for nuclear protein, the membrane was probed with anti-NF-κB-p65 subunit antibody (rabbit polyclonal, Abcam, 1:1000). The intensities of the blots were determined using an imaging analysis program (Image J, free download from http://rsbweb.nih.gov/ij/). The β-actin was used as internal control. The normalized values in different groups were averaged and expressed as fold change with the mean value of control group as 1.

### RNA extraction and quantitative RT-PCR analysis

Total RNA was extracted using TRIzol solution (Life Technologies, Inc., Rockville, MD) and then reverse-transcribed (RT) (cDNA Synthesis Kit, Bio-Rad, Hercules, CA). The RT products were amplified using a TaqMan Gene Expression Assays kit (Applied Biosystems). A kit for detecting the levels of 18S ribosomal RNA was used as an endogenous control. The relative gene expressions were calculated in accordance with the ΔΔCt method. Relative mRNA levels were expressed by the values of 2^−ΔΔCt^.

### Immunofluorescent microscopy

Cells were grown on glass chamber slides and undergone different treatments as described above. After fixation and permeabilization with 4% paraformaldehyde, cells were respectively incubated with antibodies of anti-E-cadherin, anti-α-SMA and anti-FSP-1 at 4°C overnight, followed with Alexa Fluor 555-coupled secondary antibodies at room temperature for 1 h; for NF-κB translocation measurement, cells were incubated with anti-NF-κB primary antibody, Alexa Fluor 555-coupled secondary antibodies and then followed by incubation with YO-PRO®-1 (Life Technologies) for nucleic acid staining at room temperature for 1 h. At last, stained cells were mounted and subjected to examinations using a confocal laser scanning microscope (FluoView FV1000, Olympus, Japan). These experiments were performed to observe the changes of EMT markers in renal tubular cells. Integrated optical intensity (IOD) was calculated by using an Image-Pro Plus v6.0 software (MediaCybernetics, Silver Spring, MD). The IOD values in control group were averaged, and all the IOD values were normalized to the mean value of the control group. The normalized values in different groups were averaged and expressed as fold change with the mean value of control group as 1.

### Statistics

Data were presented as means ± S.E.M. Significant differences between and within multiple groups were evaluated using an ANOVA followed by a Duncan’s multiple-range test. Student’s t-test was used to evaluate statistical significance of differences between two groups. *P*< 0.05 was considered statistically significant

## Results

### Effects of stem cell conditioned media (SCM) on albumin-induced changes in EMT markers

**Fig. 1:**
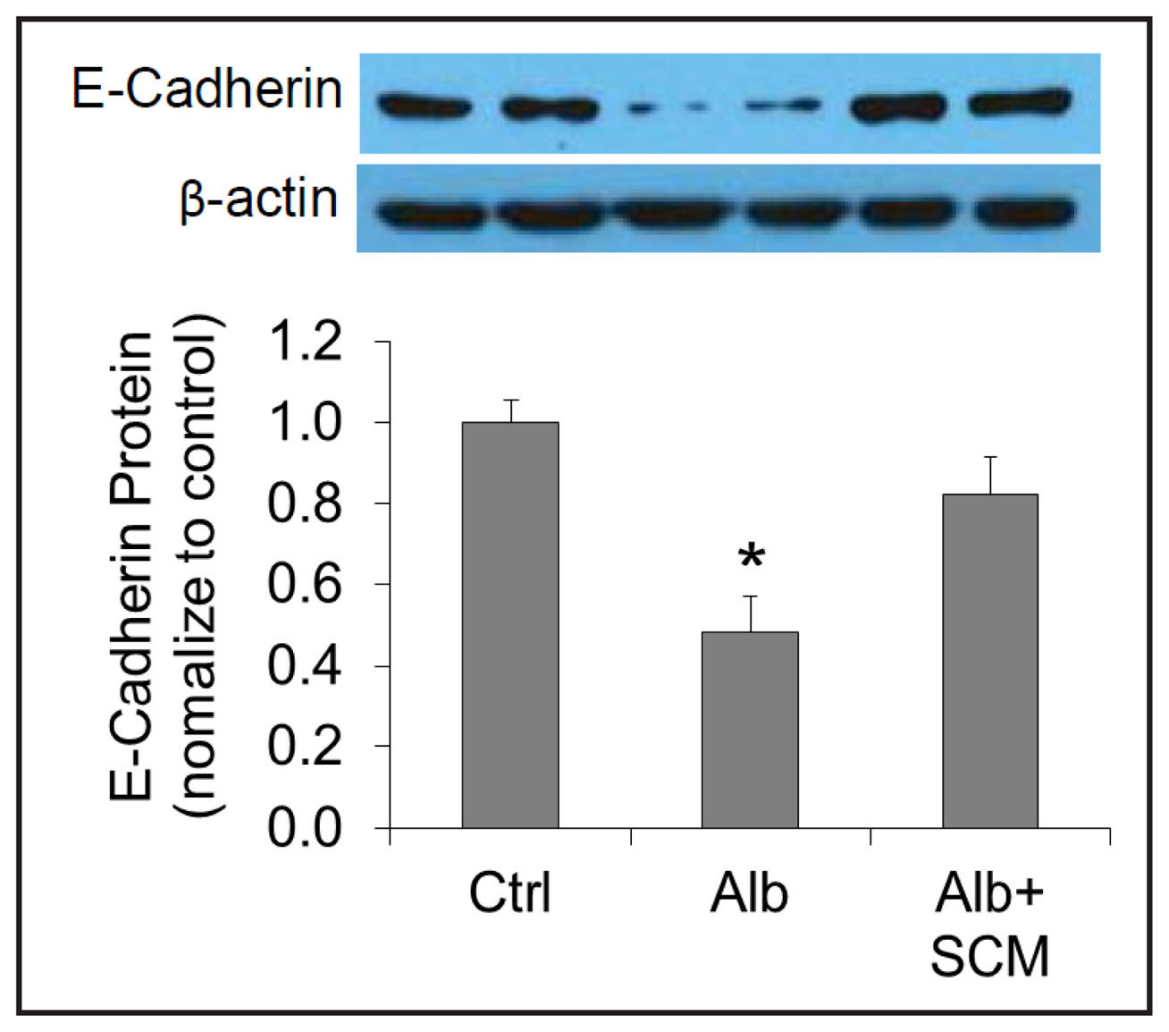
Effect of SCM on albumin-induced decrease in epithelial marker E-cadherin by Western blot analysis. Upper panel: Representative gel documents; Lower Panel: summarized data. Ctrl = control cells treated with CCM, Alb = cells treated with albumin + CCM, Alb+SCM = cells treated with albumin + SCM. n=6 batches of cells, *P<0.05 vs. other groups.

**Fig. 2:**
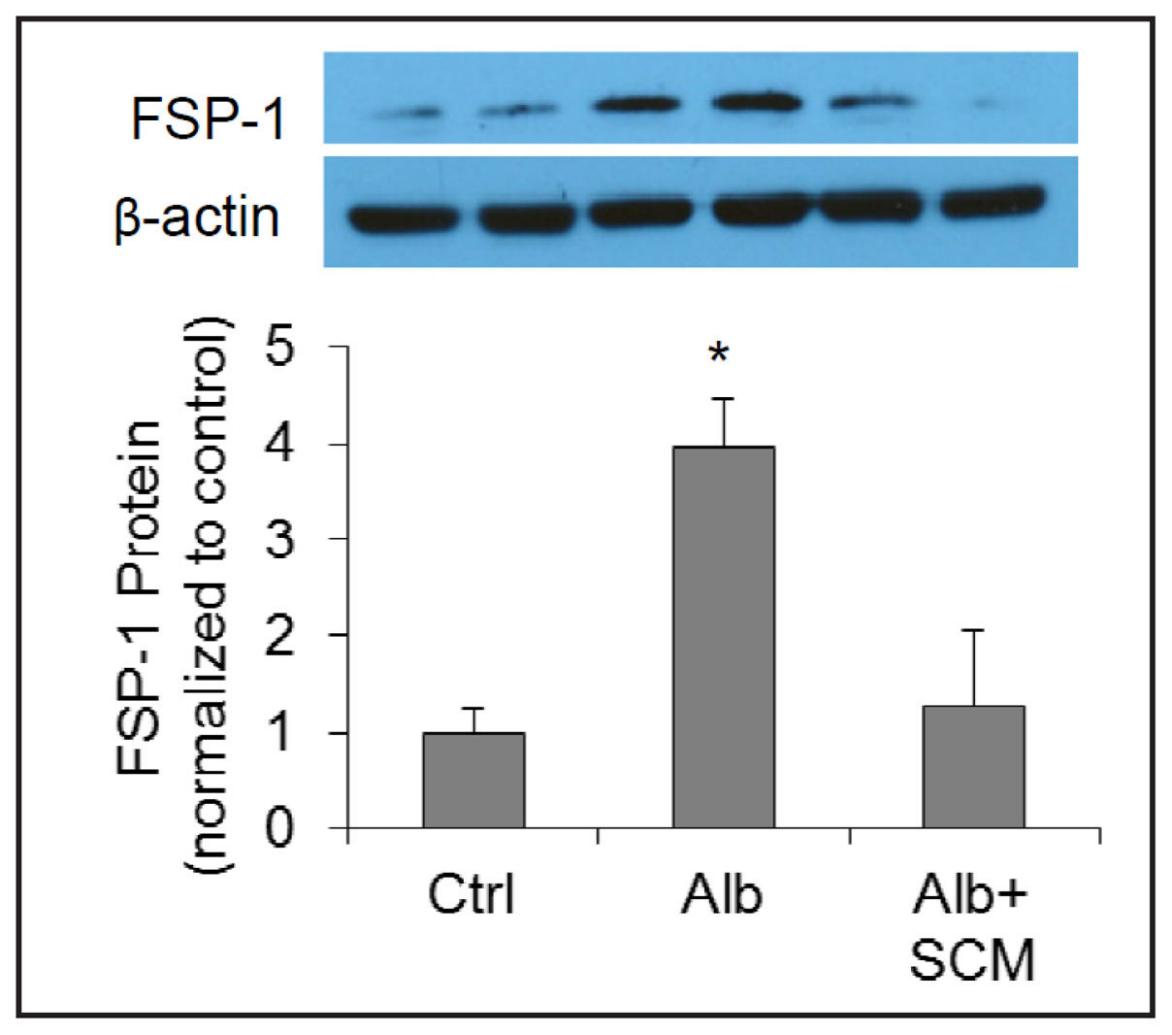
Effect of SCM on albumin-induced increase in mesenchymal marker FSP-1 by Western blot analysis. Upper panel: Representative gel documents; Lower panel: summarized data. Ctrl = control cells treated with CCM, Alb = cells treated with albumin + CCM, Alb+SCM = cells treated with albumin + SCM. n=6 batches of cells, *P<0.05 vs. other groups.

**Fig. 3:**
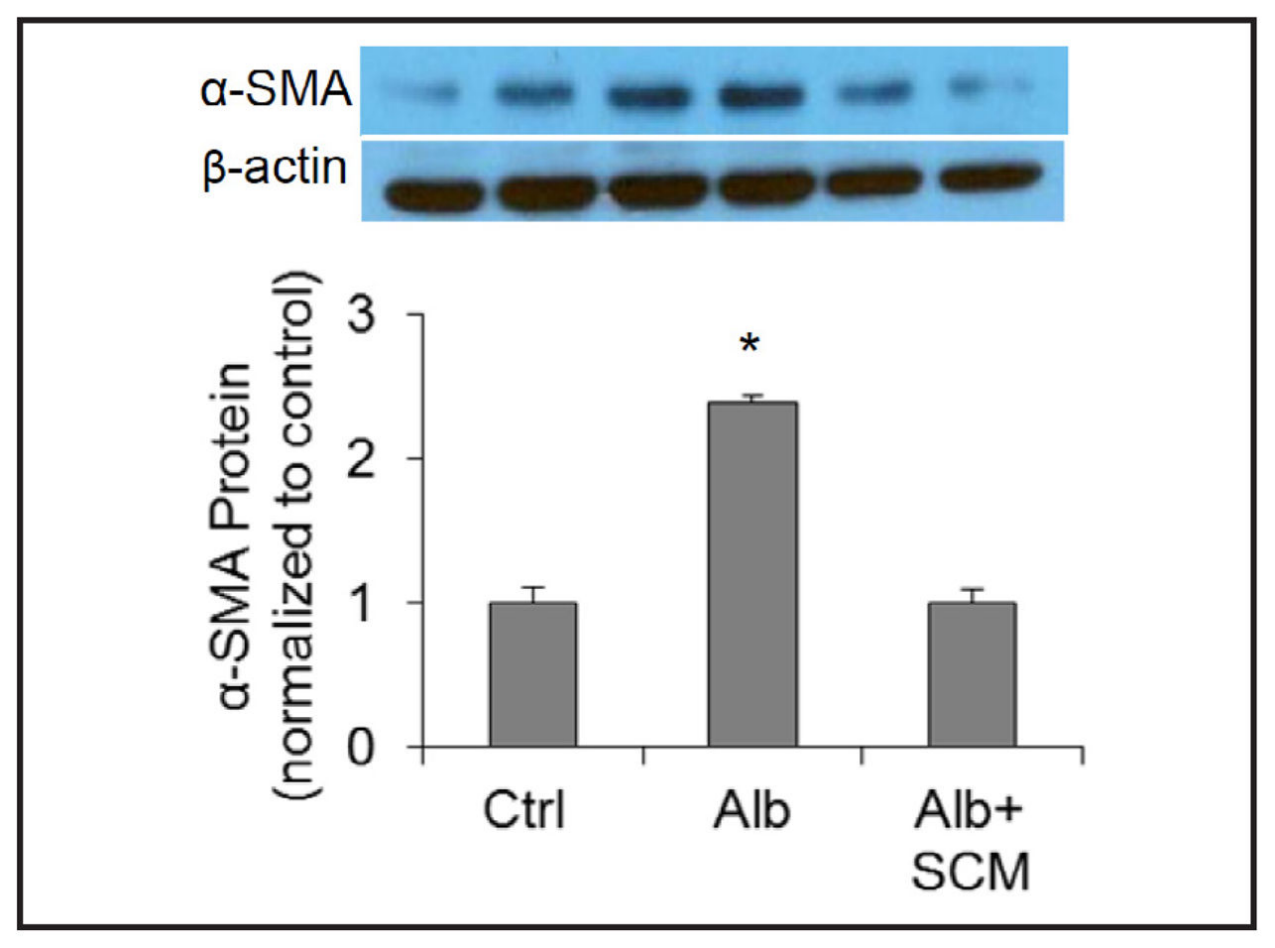
Effect of SCM on albumin-induced increase in mesenchymal marker α-SMA by Western blot analysis. Upper panel: Representative gel documents; Lower panel: summarized data. Ctrl = control cells treated with CCM, Alb = cells treated with albumin + CCM, Alb+SCM = cells treated with albumin + SCM. n=6 batches of cells, *P<0.05 vs. other groups.

Cells were treated with 1) control culture media (CCM), 2) rat albumin + CCM, and 3) rat albumin + SCM. Western blot analysis showed that protein expression of epithelial cell marker E-Cadherin was much lower in albumin-treated cells than that in control cells, whereas its expression in SCM-treated cells were recovered to levels similar to that in control cells (Fig. 1). In contrast, the protein levels of mesenchymal cell markers FSP-1 (Fig. 2) and α-SMA (Fig. 3) were both much higher in albumin-treated cells than that in control cells, whereas the levels of FSP-1 and α-SMA in SCM-treated cells were similar to that in control cells. These results indicated that albumin reduced epithelial marker and stimulated the mesenchymal markers and that SCM blocked the albumin-induced changes in EMT markers.

### Effects of SCM on the albumin-induced changes in the patterns of immunostaining in EMT markers

**Fig. 4:**
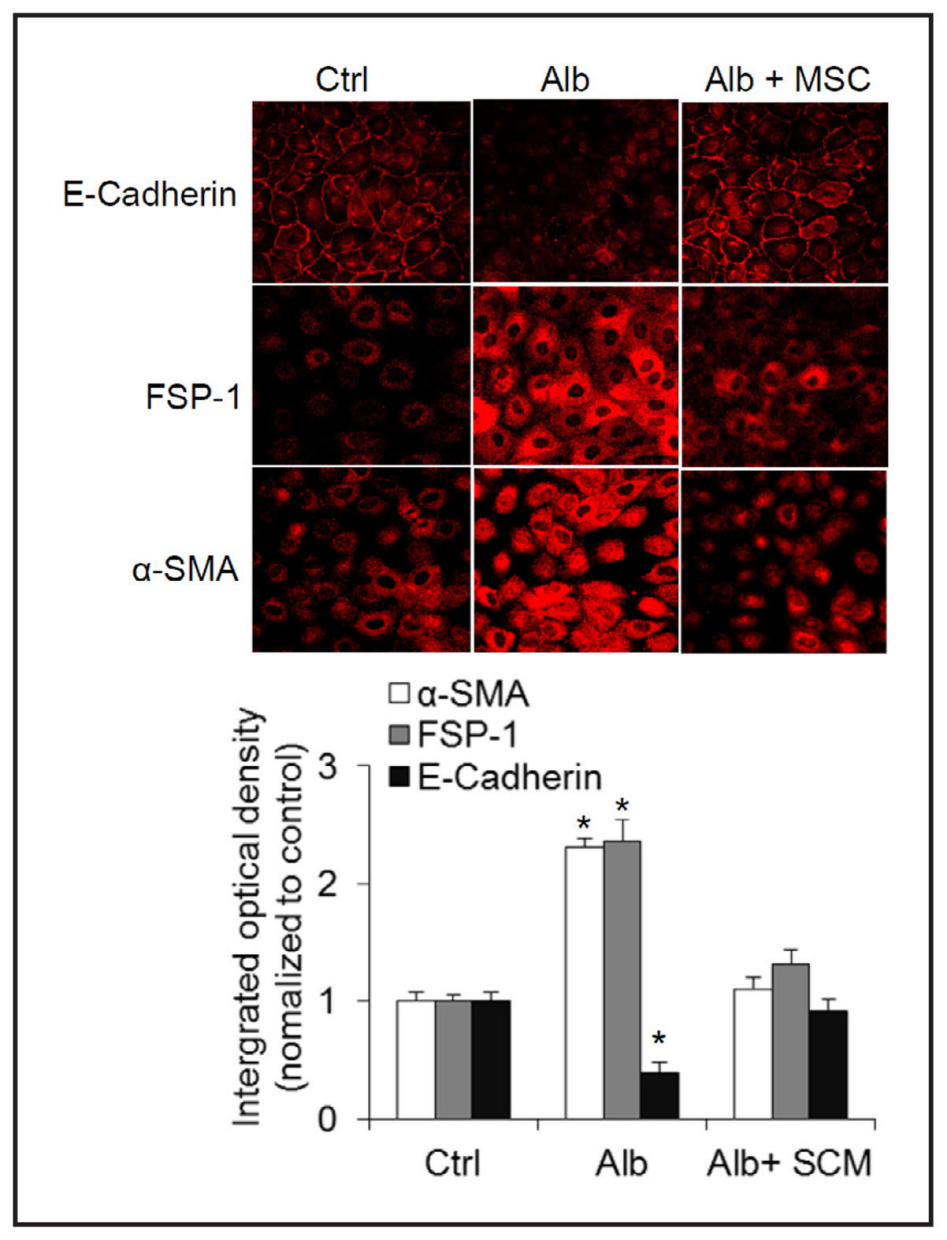
Effect of SCM on albumin-induced changes of staining patterns in E-cadherin, FSP-1 and α-SMA by immunofluorescent microscopy assay. Upper panel: Representative confocal images showing the immunostaining of E-cadherin, FSP-1 and α-SMA; Lower panel: Summarized integrated optical intensity of the fluorescent staining. Ctrl = control cells treated with CCM, Alb = cells treated with albumin + CCM, Alb+SCM = cells treated with albumin + SCM. n=5 batches of cells, *P<0.05 vs. other groups.

To further investigate the SCM effects on the albumin-induced EMT, immune staining analysis of EMT markers were performed in cells with different treatments. As shown in Fig. 4, immunostaining of E-Cadherin clearly outlined the cell contours with enriched fluorescence along cell membrane in control cells treated with CCM; in cells treated with albumin + CCM, the intensity of immunostaining was reduced and the contour of E-Cadherin staining was discontinuous; in cells treated with albumin + SCM, the contour of E-Cadherin staining was recovered and the intensity of immunostaining was significantly increased compared with that in cells treated with albumin. In contrast, the staining of mesenchymal cell markers FSP-1 and α-SMA were both weak in control cells treated with CCM (Fig. 4); in cells treated with albumin + CCM, the staining of both FSP-1 and α-SMA was much stronger than that in control cells; in cells treated with albumin + SCM, however, the staining of both α-SMA and FSP-1 was similar to the levels in control cells. These results additionally demonstrated that SCM blocked the albumin-induced changes in EMT markers.

### Effects of SCM on the albumin-induced collagen-I expression

**Fig. 5:**
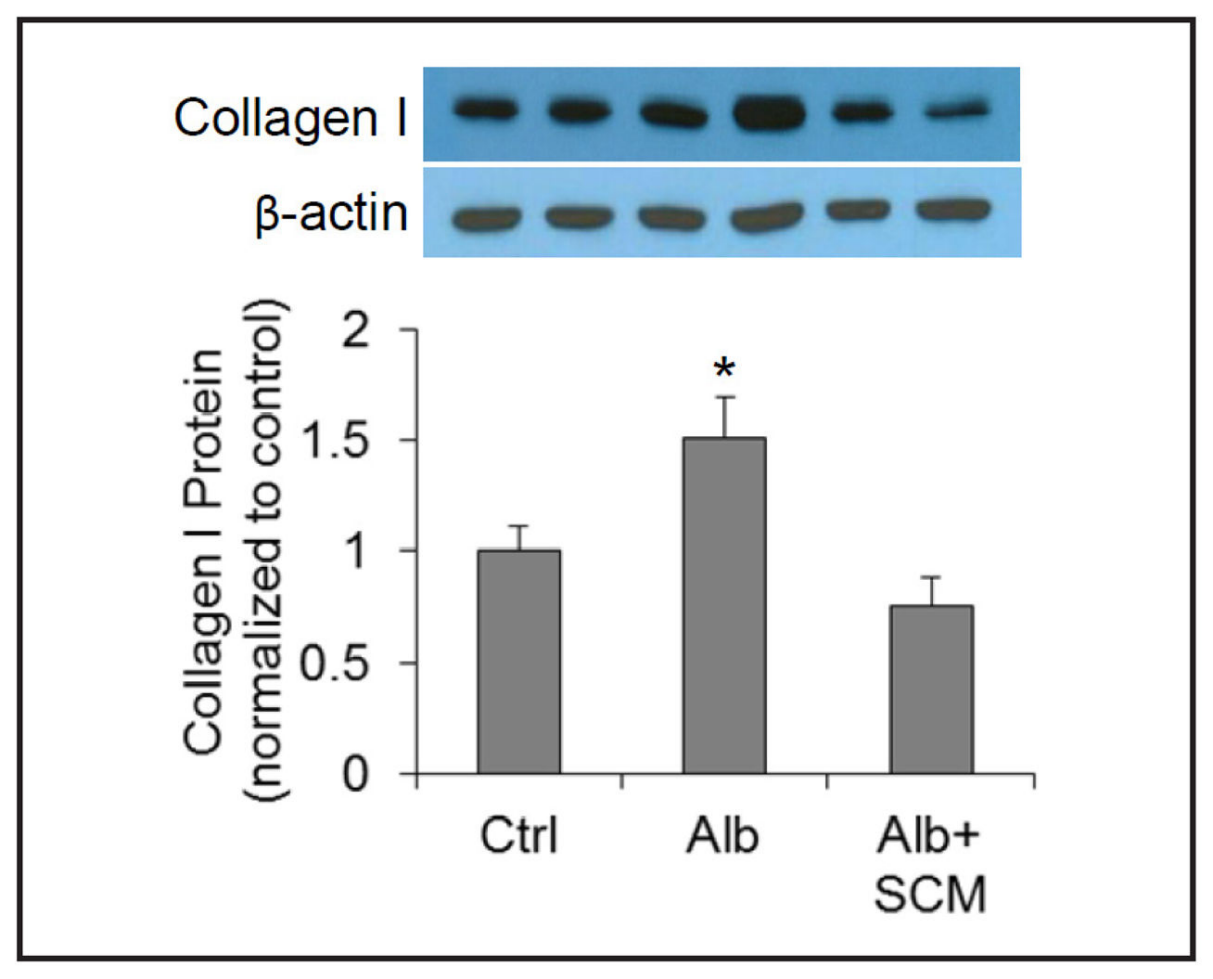
Effect of SCM on albumin-induced increase in collagen I by Western blot analysis. Upper panel: Representative gel documents; Lower panel: Summarized data. Ctrl = control cells treated with CCM, Alb = cells treated with albumin + CCM, Alb+SCM = cells treated with albumin + SCM. n=6 batches of cells, *P<0.05 vs. other groups.

The collagen-I expression was significantly higher in albumin-treated cells than in CCM-treated control cells. However, in cells treated with albumin + SCM, the levels of collagen-I protein were lower than that in albumin-treated cells, similar to that in CCM-treated control cells (Fig. 5). These data indicate that SCM inhibits albumin-induced expression of collagen-I, probably via actions on EMT.

### Effects of SCM on albumin-induced nuclear translocation of NF-κB

**Fig. 6:**
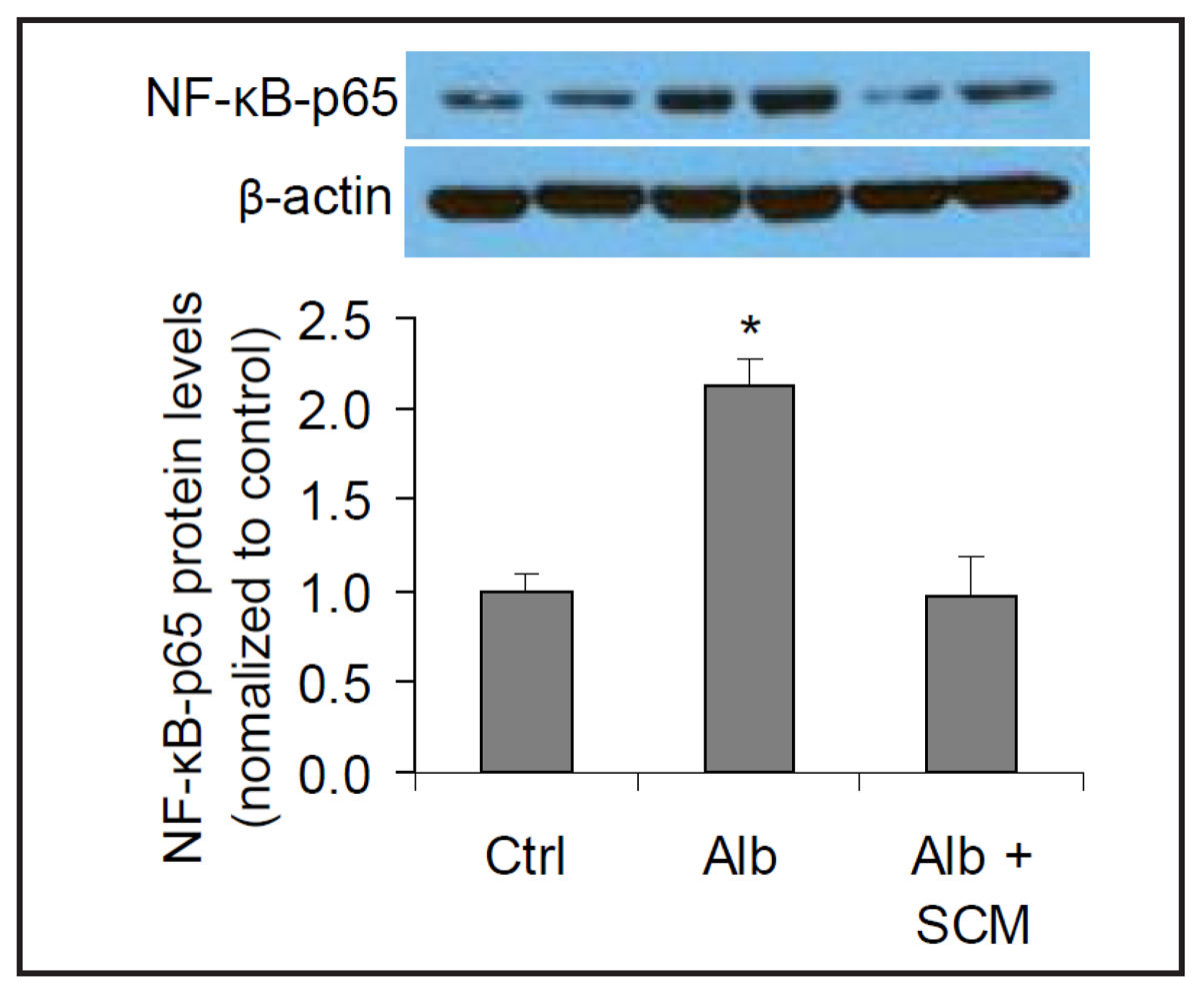
Effect of SCM on albumin-induced increase in NF-κB-p65 in nuclear proteins by Western blot analysis. Upper panel: Representative gel documents; Lower panel: Summarized data. Ctrl = control cells treated with CCM, Alb = cells treated with albumin + CCM, Alb+SCM = cells treated with albumin + SCM. n=6 batches of cells, *P<0.05 vs. other groups.

NF-κB plays a key role in regulating inflammation. It has been reported that NF-κB is involved in TGF-β-induced EMT [26]. Our Western blot results showed that NF-κB-p65 levels in nuclear protein were significantly higher in cells treated with albumin + CCM than in cells treated with CCM. However, the NF-κB-p65 levels were much lower in cells treated with albumin + SCM than in cell treated with albumin + CCM, similar to that in CCM-treated control cells (Fig. 6).

**Fig. 7:**
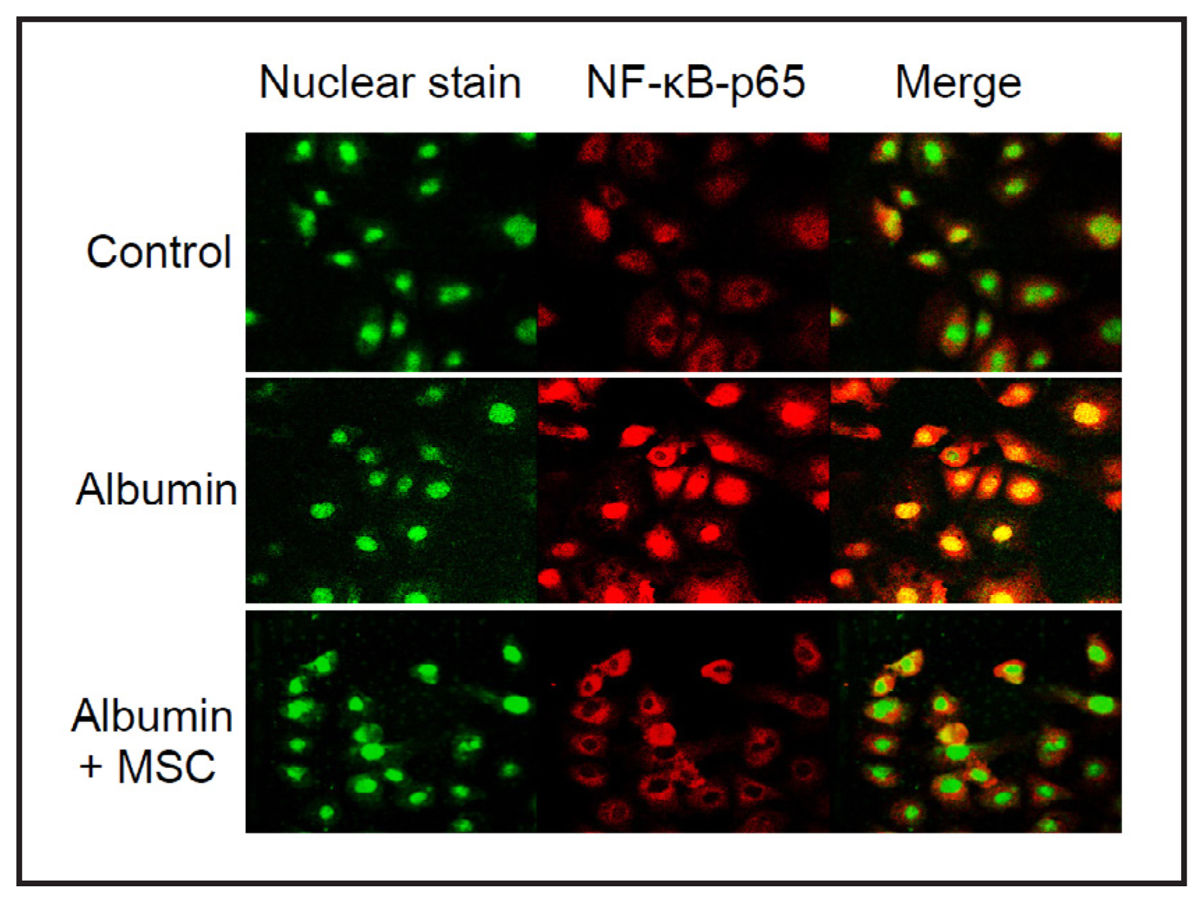
Effect of SCM on albumin-induced changes of staining patterns in NF-κB-p65 by immunofluorescent microscopy assay. Representative confocal images showing the immunostaining of nuclear marker (green) and NF-κB-p65 (red) (from 5 batches of cells). Control = control cells treated with CCM, Albumin = cells treated with albumin + CCM, Albumin+SCM = cells treated with albumin + SCM.

Effects of SCM on albumin-induced NF-κB translocation into nuclei were further investigated by fluorescence confocal images. As shown in Fig. 7, NF-κB-p65 staining was mainly located in cytoplasm with a weak staining in nuclei in most of CCM-treated control cells, whereas most of nuclei showed a strong NF-κB-p65 staining in albumin + CCM-treated cells, indicating a nuclear translocation of NF-κB. Overlaid images showed a much stronger yellow color in the nuclei in albumin-treated cells than that in control cells, further indicating a nuclear translocation of NF-κB. However, staining pattern of NF-κB-p65 in cells treated with albumin + SCM was similar to that in CCM-treated control cells. These results were consistent with the above findings by western blot assay, further suggesting that albumin-induced nuclear translocation of NF-κB were blocked by SCM treatment.

### Effects of SCM on albumin-induced expression of monocyte chemotactic protein (MCP)-1

**Fig. 8:**
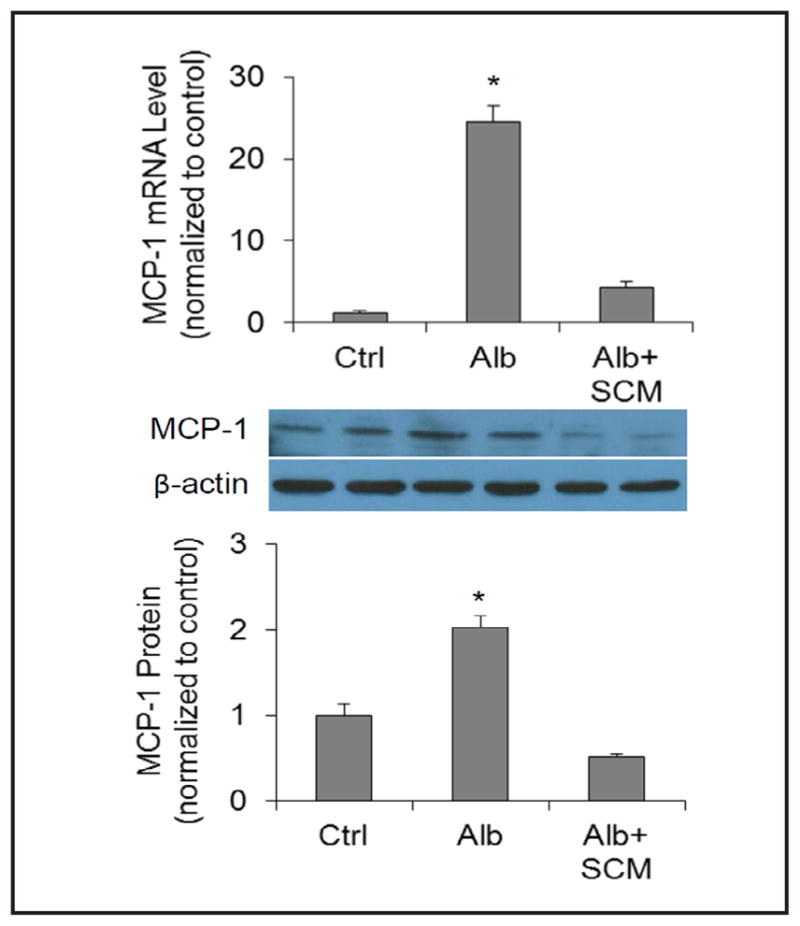
Effect of SCM on albumin-induced increase in MCP-1. Upper panel: mRNA levels by real-time RT-PCR analysis; Lower panel: Protein levels by Western blot analysis. Ctrl = control cells treated with CCM, Alb = cells treated with albumin + CCM, Alb+SCM = cells treated with albumin + SCM. n=4–6 batches of cells, *P<0.05 vs. other groups.

MCP-1 is one of the important inflammatory mediators. The levels of MCP-1 mRNA and protein expression were significantly higher in albumin-treated cells than that in control. However, in cells treated with albumin + SCM, the MCP-1 mRNA and protein expressions were much lower than that in albumin-treated cells, similar to that in CCM-treated control cells (Fig. 8). These data suggest that SCM inhibits albumin-induced activation of pro-inflammatory factors.

## Discussion

The present study demonstrated that SCM blocked albumin-induced loss of epithelial cell marker E-Cadherin and gain of mesenchymal cell markers α-SMA and FSP-1. The beneficial effects of SCM were associated with blocking the nuclear translocation of NF- κB and abolishing the increase in pro-inflammatory factor MCP-1 induced by albumin. These data indicate that SCM blocks albumin-induced EMT in renal tubular cells via anti-inflammatory actions.

Urinary protein, particularly albumin, is being recognized as a key mediator of renal tubulointerstitial injury in CKD [27]. It has been demonstrated that proteinuria is one of the most common inducer of tubular EMT [28, 29]. Given the important role of EMT in the progression of CKD, albumin-induced EMT in renal tubular cells used in this study may present a useful *in vitro* model for EMT and chronic kidney diseases.

Renal tubular epithelial cells have a low capacity of proliferation and migration. They undergo a dedifferentiation from epithelial cells into mesenchymal cells during renal injury. This dedifferentiation enables the cells to proliferate and migrate. In this dedifferentiated cells, the levels of the surface and cytoplasmic expression of α-SMA and signal transduction proteins of FSP-1, which are specially expressed on fibroblast *in vivo*, are increased [30–32], whereas the expression of epithelial makers, such as E-cadherin, is repressed [32–34]. Therefore, the reduction in the expression of epithelial cell marker E-Cadherin and elevation in the expression of mesenchymal cell markers FSP-1 and α-SMA have been widely used as indicators for EMT [32, 34, 35]. To evaluate whether SCM would have any effect on albumin-induced EMT in renal tubular cells, these EMT markers were examined in cells under different treatments in the present study. Our results showed that treatment of renal proximal tubular cells with albumin increased the expression of α-SMA and FSP-1, and suppressed E-cadherin expression, demonstrating that albumin stimulated EMT in renal tubular cells. Interestingly, in cells treated with albumin + SCM the changes in the above EMT markers were reversed, suggesting that SCM blocks albumin-induced EMT.

Synthesis of extracellular matrix molecules, mostly collagen type I and type III, is a characteristic of fibrosis. It has been shown that collagen expression is increased in epithelial cells undergoing albumin-induced EMT [11, 12]. We then detected the expression of collagen-I to further evaluate the effect of SCM on albumin-induced fibrogenesis in renal tubular cells. In addition to blocking albumin-induced changes in EMT markers, SCM also reversed albumin-induced increase of collagen-I expression, further verifying that SCM attenuated albumin-induced fibrogenic effect in renal tubular cells.

Inflammatory factors are associated with tubular EMT [36, 37]. Inflammatory factor NF-κB plays a critical role in the progression of CKD and renal fibrosis [38]. It has also been shown that NF-κB is an upstream mediator of EMT [26, 39, 40]. Inhibition of NF-κB attenuated EMT and fibrogenesis in renal tubular cells *in vivo* and *in vitro* [41]. We therefore determined whether SCM acted on NF-κB. Our results showed that albumin induced an activation of NF-κB, as indicated by the increased levels of NF-κB in nuclear protein and translocation of NF-κB from cytoplasm into nucleus, whereas SCM blocked albumin-induced NF-κB translocation from cytoplasm into nucleus. These data suggest that SCM attenuates albumin-induced EMT in renal tubular cells via inhibiting the activation of inflammatory factor NF-κB. Our findings are consistent with previous reports that stem cells and SCM possess anti-inflammatory prosperities [42–44].

Proteinuria can stimulate renal tubular cells to produce inflammatory factors that recruit immune cells to further produce EMT and damage the cells *in vivo*. MCP-1 represents one of such important cytokines to recruit immune cells and cause inflammation *in vivo*. Our results showed that SCM inhibited albumin-induced increase of MCP-1, further indicating that SCM acted on pro-inflammatory effects of albumin in renal tubular cells. On top of directly targeting tubular cells to inhibit EMT, SCM would possess additional anti-inflammatory function *in vivo* to target the mobilization of immune cells by inhibiting pro-inflammatory factors, such as MCP-1, which would inhibit inflammation and further attenuate EMT *in vivo* in CKD.

It should be pointed out that there remain questions derived from the data in the present study. The present study just detected NF-κB and MCP-1 as representative samples associated with albumin-induced pro-inflammatory effects. Many other factors are also involved in albumin-induced EMT. SCM may act on other factors that mediate EMT as well. For example, TGF-β plays an important role in the progression of CKD [45] and that protein loading stimulates TGF-β in renal cells [7]. Whether SCM also inhibits TGF-β and other factors associated with CKD is worth investigating in the future studies. In addition, SCM contains a large number of various growth factors and cytokines that may contribute to the beneficial effects of SCM [46, 47]. What factors in SCM are responsible for the effects observed in the present study remain unanswered. This important and rather complicated question requires further investigation [46]. Furthermore, the findings in the present *in vitro* study require confirmation *in vivo*. It has been shown that intravenous or local injection of SCM improves different disease conditions [46, 48–50]. Similar approaches to examine the effect of SCM on the progression of renal diseases using *in vivo* models with proteinuria needs to be performed in the future.

In summary, the present study demonstrated that SCM attenuated albumin-induced EMT, which was associated with inhibition of albumin-induced increase of pro-inflammatory factors and the activation of NF-κB. SCM or factors released in the SCM may serve as an approach to slow the progression of CKD by preventing the proteinuria-induced pro-inflammatory factors and EMT. The results from the present study may also indicate that the functions of adult stem cells in the kidneys may constitute one of the mechanisms counteracting proteinuria-induced inflammation and EMT in CKD.
